# Immunotherapy for Bone and Soft Tissue Sarcomas

**DOI:** 10.1155/2015/820813

**Published:** 2015-06-17

**Authors:** Takenori Uehara, Tomohiro Fujiwara, Ken Takeda, Toshiyuki Kunisada, Toshifumi Ozaki, Heiichiro Udono

**Affiliations:** ^1^Department of Orthopaedic Surgery, Okayama University Graduate School of Medicine, Dentistry and Pharmaceutical Sciences, Okayama 700-8558, Japan; ^2^Department of Immunology, Okayama University Graduate School of Medicine, Dentistry and Pharmaceutical Sciences, Okayama 700-8558, Japan; ^3^Center of Innovative Medicine, Okayama University Hospital, Okayama 700-8558, Japan; ^4^Department of Intelligent Orthopaedic System, Okayama University Graduate School of Medicine, Dentistry and Pharmaceutical Sciences, Okayama 700-8558, Japan; ^5^Department of Medical Materials for Musculoskeletal Reconstruction, Okayama University Graduate School of Medicine, Dentistry and Pharmaceutical Sciences, Okayama 700-8558, Japan

## Abstract

Although multimodal therapies including surgery, chemotherapy, and radiotherapy have improved clinical outcomes of patients with bone and soft tissue sarcomas, the prognosis of patients has plateaued over these 20 years. Immunotherapies have shown the effectiveness for several types of advanced tumors. Immunotherapies, such as cytokine therapies, vaccinations, and adoptive cell transfers, have also been investigated for bone and soft tissue sarcomas. Cytokine therapies with interleukin-2 or interferons have limited efficacy because of their cytotoxicities. Liposomal muramyl tripeptide phosphatidylethanolamine (L-MTP-PE), an activator of the innate immune system, has been approved as adjuvant therapeutics in combination with conventional chemotherapy in Europe, which has improved the 5-year overall survival of patients. Vaccinations and transfer of T cells transduced to express chimeric antigen receptors have shown some efficacy for sarcomas. Ipilimumab and nivolumab are monoclonal antibodies designed to inhibit immune checkpoint mechanisms. These antibodies have recently been shown to be effective for patients with melanoma and also investigated for patients with sarcomas. In this review, we provide an overview of various trials of immunotherapies for bone and soft tissue sarcomas, and discuss their potential as adjuvant therapies in combination with conventional therapies.

## 1. Introduction

Sarcomas are malignant tumors of mesenchymal origin, including bones, muscles, fat, nerves, and blood vessels. According to the Surveillance Epidemiology and End Results (SEER) database, prevalence of sarcoma accounts for nearly 21% of all pediatric solid malignant tumors and less than 1% of all adult solid malignant tumors [1]. It was estimated that approximately 11,400 Americans would be diagnosed with soft tissue sarcomas and 3,000 with bone sarcoma in 2013 [2]. Based on the survival data obtained from the National Cancer Data Base of the American College of Surgeons, the relative 5-year survival rate is approximately 66% for patients with bone and soft tissue sarcomas, 53.9% for osteosarcomas (*n* = 8,104), 75.2% for chondrosarcoma (*n* = 6,476), and 50.6% for Ewing's sarcomas (*n* = 3,225) [3]. According to the classification by the World Health Organization, the group of bone and soft tissue sarcomas includes more than 100 histological subtypes [4]. The prognosis of patients with bone and soft tissue sarcomas is associated with histological diagnoses [5]. Standard treatment modalities include surgical resection, chemotherapy, and often radiotherapy [6–8]. Despite these multimodality therapies, survival rates have not been improved over recent 20 years [9]. Therefore, new effective treatment over conventional therapy is urgently needed.

Historically, Coley reported a case of unresectable small-cell sarcoma of the neck in 1891. The sarcoma completely regressed after a severe episode of erysipelas. He reported that a systemic response against erysipelas influenced the patient's tumor [10]. The mechanism by which erysipelas caused tumor regression was unclear at that time. However, it is now understood that the activation of innate immunity through Toll-like receptors (TLRs) by erysipelas followed by activation of acquired immunity specific to sarcoma may contribute to the underlying mechanism [11]. Thus, the case described by Coley was the first to demonstrate that the immune system is involved in the spontaneous regression of sarcomas. Over the past 100 years, his work had encouraged many scientists to work on cancer immunology, in an attempt to find a cure for cancers [12, 13].

The dissection of the molecular mechanisms of innate and acquired immunity has enabled medical doctors and scientists to apply various cancer immunotherapies such as vaccines, antibodies, adjuvants, and cell therapies [29–31]. Utilizing modern cancer immunotherapies for patients with sarcomas began in the 1980s as a cytokine therapy [32, 33], and more recently antigen-specific cancer vaccines and/or cell therapies have been developed [34, 35].

## 2. Overview of Cancer Immunology

### 2.1. Immune System Overview

Knowledge about the immune system is essential for understanding the principles underpinning cancer immunotherapy. There are two types of immune responses against microbes: called innate and adaptive immunity [36]. Innate immunity, whose main components are phagocytic cells (neutrophils and macrophages) and natural killer cells, provides the initial defense against invading microbes during infection [37, 38]. Small molecular proteins called cytokines mediate many activities of the cells involved in innate immunity. In addition to cytokines, pattern recognition molecules such as TLRs expressed on dendritic cells (DCs) and macrophages play critical roles in the activation of innate immunity. These components also have a role in communicating with acquired (adaptive) immunity [39, 40]. The key components of adaptive immunity, following the initial innate immunity, are T and B lymphocytes. The lymphocytes play a central role in eliminating infectious pathogens, virus infected cells, and cancer cells and also in generating antigen-specific memory cells [37].

Adaptive immunity consists of humoral and cell-mediated immunity. T lymphocytes recognize short peptides as antigens presented by major histocompatibility complexes (MHCs) on the cell surface of DCs [41, 42]. CD8 and CD4 T cells recognize antigen in the context of MHC class I and class II molecules, respectively [43, 44]. Primed and activated T cells differentiate into mature effector cells while undergoing clonal expansion. The effector CD8 T cells recognize virus infected cells and tumor cells and eliminate them from the body. The differentiation of naïve CD8 T cells into effector and memory CD8 T cells is mediated by the “help” of CD4 T cells or by a stimulation of TLRs of DC [43–45]. “Help” means signals occurring within DCs whose CD40 interacts with CD40L of CD4 T cells to express large amounts of CD80/86 to interact with CD28 of CD8 T cells [46–48]. Signals from either CD40 or TLRs activate DCs, and this process then initiates the activation of naïve CD8 T cells following antigen recognition [49].

DCs, B cells, and macrophages are professional antigen-presenting cells (APCs) [50, 51]. Among them, DCs are the most effective APCs [51, 52]. For example, B cells and macrophages present endogenous and internalized exogenous antigens with MHC class I and class II molecules [53], respectively. Therefore, B cells and macrophages can only activate CD4 T cells when they internalize extracellular antigens [54]. On the other hand, DCs are able to process both endogenous and exogenous antigens with MHC class I molecules to activate CD8 T cells. This is referred to as cross-presentation and is essential in fighting against virus infected cells and tumor cells [55–57].

### 2.2. Tumor Immunology and Immune Checkpoint

**Figure 1:**
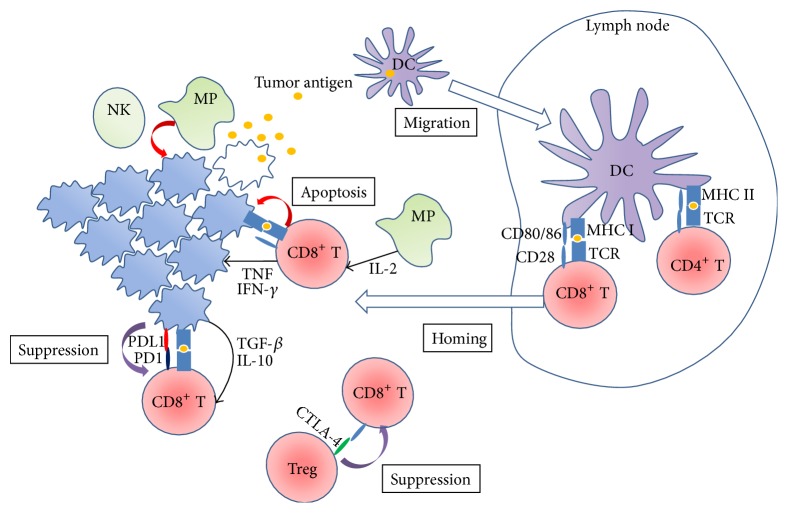
An overview of tumor immunology. Tumor cells are initially attacked by the innate immune system. DCs capture tumor antigens at the tumor site and migrate to the tumor draining lymph nodes. DCs present the tumor antigen to T cells within the lymph node. Antigen-specific CD4 and CD8 T cells are stimulated by DCs. After stimulation, T cells differentiate into effector cells and activate at the tumor site. Effector CD8 T cells kill tumor cells, although their function is regulated by the immune checkpoint mechanism. NK: natural killer cell; MP: macrophage; DC: dendritic cell.

Tumor antigens recognized by the immune system are categorized into cancer testis antigens (CTAs), melanocyte differentiation antigens, mutated proteins, overexpressed proteins, and viral antigens [58] (Figure 1). Several types of CTAs have been identified in patients with sarcomas (Table 1). Because tumor antigens are potential targets that induce cytotoxic immune responses [59], many clinical trials have utilized tumor antigens as vaccines for decades. The results, however, are limited and the desired therapeutic effect is not achieved [60, 61].

Although antitumor immunity is induced in patients with cancer vaccines, recent advancements in cancer immunity have revealed the presence of immune-inhibitory mechanisms, referred to as immune checkpoints [62], in the draining of lymph nodes and tumor sites. CTLA-4, a protein receptor expressed on T cells, downregulates T cell activation [63]. The structure of CTLA-4 is similar to CD28, with a T cell costimulatory receptor. Immune inhibition is caused by the competition between CD28 and CTLA-4 to bind CD80/86 on DCs [64]. Regulatory T cells (Tregs) that define CD4^+^CD25^+^Foxp3^+^ T cells highly express CTLA-4 and suppress the activation of cytotoxic lymphocytes [65]. The inhibition of activated T cells via CTLA-4 occurs particularly within draining lymph nodes [66]. Programmed cell death protein 1 (PD1) is also an immune checkpoint receptor expressed on T cells, particularly cytotoxic lymphocytes [67, 68]. Tumor cells upregulate the expression of PD-ligand 1(PD-L1), and the interaction of PD1 with PD-L1 downregulates the function of T cells within the tumor microenvironment [69, 70]. The immune checkpoint is therefore considered to be an important therapeutic target. Anti-CTLA-4 and anti-PD1 antibodies have been introduced for clinical use in some cancers [71]. In addition to CTLA4 and PD-1, there are similar cell surface molecules of activated effector T cells, such as Tim-3 and LAG3, that suppress tumor immunity [72]. Inflammation in the tumor microenvironment induces STAT3 activation within tumors and Tregs. In contrast, STAT3 in certain tumors is constitutively activated by genetic alterations [73, 74]. STAT3 activation leads tumor cells and Tregs to express molecules that are related to immune checkpoints, such as PD-L1, and eventually inhibit T cell function [75, 76].

## 3. Outcomes of Clinical Trials for Bone and Soft Tissue Sarcomas

Treatments for bone and soft tissue sarcomas include surgery, chemotherapy, and radiotherapy. To date, clinical results of combined therapies have been more successful than those of surgical approaches. However, as described above, the prognosis of bone and soft tissue sarcomas has plateaued since the 1990s. In these recent years, immunotherapies are expected to further improve the prognosis of patients, and several clinical trials have been performed (Tables 2 and 3).

### 3.1. Cytokine Therapies

Cytokines are proteins that regulate the immune system. Interleukin-2 (IL-2) and interferons (IFNs) have been used in the immunotherapy for sarcomas [77], and clinical results are evident. IL-2 leads to the activation and expansion of CD4 and CD8 T cells [78]. Rosenberg et al. established a tumor regression model involving recombinant IL-2 injection for murine melanoma and sarcomas [32]. Then, several studies described the effectiveness of high-dose IL-2 therapy for patients with metastatic melanomas [79, 80]. Therefore, recombinant IL-2 was administered to patients with bone and soft tissue sarcomas [16]. Schwinger et al. reported a positive clinical result using a high-dose IL-2 treatment in two patients with Ewing's sarcomas and four patients with metastatic osteosarcomas. Patients had already been treated with surgery (1–5 times), chemotherapy (7–43 cycles), and radiation therapy (for patients with Ewing's sarcoma). Although one patient with metastatic osteosarcoma progressed during the treatment period, two patients with osteosarcoma achieved complete responses with a median follow-up time of 28 months (range: 11–36 months). However, all patients experienced adverse effects such as fatigue, anorexia, diarrhea, nausea, vomiting, and high-grade fever. Two patients could not undergo IL-2 therapy [16]. Furthermore, the other initial study reported treatment related death caused in 1-2% of patients [81]. Consequently, it limited the administration of high-dose IL-2 therapy for its adverse effect [81, 82].

The use of IFN-*α* as an adjuvant therapy was initiated at the Karolinska Hospital in 1971 [19]. The Karolinska Hospital group reported that 10-year results of adjuvant IFN-*α* therapy. The clinical outcome was improved by introducing adjuvant IFN-*α* therapy. The metastasis-free survival rate was 39% and the sarcoma-free survival rate was 43% in adjuvant IFN therapy group. These clinical results were better than the group of surgiral therapy only (15–20%) [83]. COSS-80 study investigated the effectiveness of use of adjuvant chemotherapy with IFN [20]. The 30-month disease-free survival rate of the IFN arm was 77% and that of non-IFN arm 73%. However, there was no significant difference between two groups; EURAMOS-1 study, a recent study in Europe, investigated the efficacy of the use of adjuvant chemotherapy with pegylated-IFN*α*-2b [21]. In the interim statement, the median follow-up time in EURAMOS-1 study was 3.1 years. The event-free survival rate was 77% in the group with chemotherapy and IFN and 73% in the group without IFN [19]. This difference was also not significant. These observations suggest that conventional chemotherapy with IFN improves the prognosis of bone and soft tissue sarcomas to some extent.

### 3.2. Mifamurtide

Mifamurtide, liposomal muramyl tripeptide phosphatidylethanolamine (L-MTP-PE), is a new agent that is a synthetic analog of a muramyl dipeptide (MDP) [22]. Although its pharmacological behavior is similar to that of MDP, L-MTP-PE has a longer half-life than MDP [84]. The intracellular pattern recognition molecule NOD2 detects MDP and enhances NF-*κ*B signaling [85]. Therefore, recognition of L-MTP-PE by NOD2 stimulates the production of IL-1*β*, IL-6, and TNF-*α* via the activation of NF-*κ*B signaling in monocytes and macrophages [86, 87].

The efficacy of L-MTP-PE treatment for osteosarcomas has been examined in dogs. Dogs with postoperative osteosarcomas were treated by intravenous L-MTP-PE injections. The median survival time of dogs treated by L-MTP-PE (222 days) was longer than that of nontreated dogs (77 days) [88]. In human, intergroup study 0133 (INT 0133) began in 1993. 662 patients with osteosarcoma were recruited in this study. The aim of the study was to evaluate the efficacy of supplementation with ifosfamide (IFO) and L-MTP-PE in basic adjuvant chemotherapy (cisplatin, doxorubicin, and high-dose methotrexate (MAP)). Patients were randomly assigned to receive MAP alone, MAP + IFO, MAP + L-MTP-PE, and MAP + IFO + L-MTP-PE. It was observed that the addition of L-MTP-PE to chemotherapy improved the six-year overall survival rate from 70% to 78% (*P* = 0.03). The hazard ratio for overall survival with the addition of MTP was 0.71 (95% CI: 0.52–0.96) [22, 89]. Therefore, L-MTP-PE has been approved in Europe for the treatment of osteosarcoma with chemotherapy. However, it has not been approved by FDA in the United States [87].

### 3.3. Vaccines

Multiple clinical trials using vaccines that target whole cells, lysates, proteins, and peptides have been investigated in patients with sarcomas [90–92]. Vaccines are combined with costimulatory adjuvants such as GM-CSF or IL-2 to enhance the immune response [93]. Therapeutic tumor vaccines are presented as antigen epitopes on MHC molecules by APCs. Tumor antigen specific T cells are activated by APCs. The aim of cancer vaccines is to stimulate the patient's own immune system to eliminate the tumor [94].

Autologous sarcoma cell lysates can be used as a vaccine in patients with sarcomas. A clinical study was performed to treat patients using their autologous tumor cell lysate as vaccines [23]. The study recruited 86 patients with sarcomas and tryed to establish short-term cell lines in vitro. 25 patients, who had an established tumor cell line, were injected with the tumor lysate vaccine. Before vaccine treatment, patients were screened to ensure they were not positive for delayed-type hypersensitivity (DTH) to irradiated tumor cells. After treatment, eight patients became positive for DTH. The median survival time of patients who became positive for DTH (16.6 months) was eight months longer than that of DTH-negative patients (8.2 months). However, objective responses were not recorded [23]. In the result, tumor lysate vaccines improved the survival time, but tumor regression disappeared.

Autologous DCs that are pulsed ex vivo with tumor cell lysate can stimulate host antitumor immunity [95, 96]. Adjuvant therapies using tumor lysate-pulsed DCs were investigated for children with solid tumors including bone and soft tissue sarcomas. After tumor lysate-pulsed DC transfer, 70% of patients changed positively in the DTH test. This study resulted in one patient achieving complete remission and in five patients, the disease stabilized during the follow-up period of 16–30 months [97].

Tumor specific or overexpressed peptides are possible for therapeutic targets for antigen-specific immunotherapy [98, 99]. Bone and soft tissue sarcomas can have specific gene mutations and express mutated proteins [100]. Synovial sarcomas are known to have chromosomal translocation and synthesize the SYT-SSX mutated protein [101]. Kawaguchi et al. treated patients who had synovial sarcomas with SYT-SSX fusion gene-derived peptides [102]. The study enrolled 21 patients, who were injected subcutaneously with the 9 mer peptide with or without incomplete Freund's adjuvant (IFA) and IFN-*α*. Nine patients were injected with the peptide alone, and later in the study, 12 patients were injected the peptide with IFA and IFN-*α*. After treatment, in seven patients, the peptide tetramer-positive CD8 T cells appeared in PBMCs. With regard to the clinical result, in six patients, the disease stabilized during vaccination; however, in other patients, the disease progressed [26].

Tumor antigen-specific peptide pulsed DCs can stimulate peptide specific T cells 150 times more efficient than peptide alone [103]. Tumor-specific peptide pulsed DCs have been administered for immunotherapy against sarcoma, leukemia, and glioma [104]. 30 patients with Ewing's sarcomas and alveolar rhabdomyosarcoma were enrolled in a study for consolidative therapy. Patients were separated into three cohorts that received different dose of IL-2 (high, low, and none). Monocyte-derived DCs were cultured with tumor-derived breakpoint peptides (EWS-FLI1, EWS-FLI2, and PAX3/FKHR), and the E7 peptide was used as control [24]. After treatment, 39% of patients generated immune responses to the vaccinating peptide. The five-year overall survival of the immunotherapy group was 43% and that of the no-immunotherapy group was 31% [24]. Further, this treatment showed no severe adverse effect. For these reasons, vaccines from tumor cell lysate or tumor specific peptide can activate adaptive immune response against tumors. Antigen-specific peptide pulsed DCs can also enhance immune response. Vaccine therapies have validity for bone and soft tissue sarcomas.

CTAs are expressed only in germ line cells in humans; however, they are also expressed in various tumors [105]. More than 40 antigens have been identified [105]. For example, NY-ESO-1 is expressed in many osteosarcomas, leiomyosarcomas, and synovial sarcomas and LAGE-1 is expressed in liposarcomas, leiomyosarcomas, and synovial sarcomas (Table 1) [106]. MAGE-A3 was administered to patients with stage III/IV melanoma [107]. The effectiveness of MAGE-A3 against non-small-cell lung cancer (NSCLC) was reported in a phase II clinical trial [108, 109]. Thus CTAs have a potential to be immunotherapeutic targets against bone and soft tissue sarcomas.

### 3.4. Adoptive Cell Transfer

Adoptive cell transfer therapy is considered to provide large number of tumor reactive CD8T cells that secrete high levels of cytokines, IFN*γ*, TNF*α*, and IL-2 [110]. Tumor infiltrating lymphocytes (TILs) include tumor reactive CD8T cells. Antigen-specific T cells were sorted from patients. T cells were expanded and stimulated ex vivo. After ex vivo treatment, activated effector T cells were transferred to patients [110]. A small study examined six patients with synovial sarcomas or metastatic melanomas expressing NY-ESO-1. For inducing tumor lysis, T cell receptor (TCR) gene-modified T cells redirected towards NY-ESO-1 were generated [28]. Modified TCR displayed T cells were expanded with IL-2 ex vivo and then transferred to patients [111]. Two patients with melanoma showed complete regression, and 1 patient with synovial sarcoma showed disease stabilization for 18 months. Some types of adoptive cell transfer therapies are ongoing for patients with sarcomas, including autologous DC transport therapy for soft tissue sarcomas (NCT01347034) and hematopoietic cell transplantation and natural killer cell transport therapies for Ewing's sarcomas and rhabdomyosarcomas (NCT02100891).

### 3.5. Immune Checkpoint Blockade

Immune checkpoint blockade is likely to advance anticancer immunology. Ipilimumab, a fully human monoclonal antibody (IgG1), blocks CTLA-4 and promotes antitumor immunity [112]. Patients with metastatic melanomas treated with ipilimumab showed improved overall survival (from 6.4 months to 10.0 months) [113]. Six patients with advanced synovial sarcoma enrolled in a phase II study were treated with ipilimumab. The overall survival time ranged from 0.77 to 19.7 months (median: 8.75 months). Immunological responses after the treatment were different in each patient, and three patients showed an enhanced titer of CT24 (an uncharacterized CTA). All sarcomas expressed NY-ESO-1; however, NY-ESO-1 titers did not show any remarkable change [114].

Another immune checkpoint blockade agent is a human monoclonal anti-PD-1 antibody, called nivolumab [115]. Nivolumab has demonstrated efficacy against several types of cancers including melanoma, NSCLC, prostate cancer, renal cell carcinoma, and colorectal cancer [116]. The reported clinical outcomes of nivolumab therapies include a cumulative response rate of 18% among patients with NSCLC, 28% among patients with melanoma, and 27% among patients with renal cell carcinoma [116]. Furthermore, a phase I trial of nivolumab combined with ipilimumab enrolled 53 patients with advanced melanoma. This trial reported that 53% of patients experienced grade 3 or 4 adverse effects related to the therapy and 53% of patients had an objective response. Among patients treated with ipilimumab as a control, 20% had an objective response [117]. Thus, immune checkpoint blockade agents demonstrate efficacy in some types of tumors; however, further information is required to confirm the effectiveness of the immune blockade agents ipilimumab and nivolumab for bone and soft tissue sarcomas.

## 4. Conclusion and Future Directions

Conventional treatment for bone and soft tissue sarcomas consists of surgical resection, chemotherapy, and radiotherapy. However, clinical outcomes by these therapeutic modalities have not significantly improved in recent decades. Under these circumstances, immunotherapy is expected to be a new therapeutic option for treatment. Cytokine therapies were initially regarded as a form of immunotherapy; however, their effectiveness was limited because of their toxicities. Only IFN-*α*-2 is used for maintenance therapy. Although L-MTP-PE induces antitumor effects via macrophage activation, the FDA has not approved its use because of the limited effectiveness. In Europe, L-MTP-PE efficacy has been confirmed in an international multicenter study. Vaccine therapy using tumor lysates or lysate-derived DCs has been investigated only in small-scale studies and in nonsarcoma patients. CTA peptide and fusion protein peptide therapies are expected to be novel sarcoma-effective vaccines. Addition of L-MTP-PE as an adjuvant may improve the vaccine therapy outcome. Novel microparticle-based drug delivery systems, such as microemulsion, nanoemulsion, nanoparticles, liposomes, and others, can load many kinds of various drugs and improve the drug delivery to target sites [118–121]. It has been reported that these systems improve the efficacy of vaccine and reduce adverse effects of cytokines [122–124]. Tuftsin, a tetrapeptide (Thr-Lys-Pro-Arg) fraction of immunoglobulin G molecule, binds to neutrophils and macrophages [125–127]. Tuftsin stimulates their phagocytic activity and enhances expression of nitric oxide synthase in macrophages. It has been demonstrated that tuftsin improves the efficacy of antibiotics against protozoan, bacterial, and fungal infections. Besides, tuftsin-bearing liposomized etoposide enhanced the therapeutic efficacy in murine fibrosarcoma models [128].

Immune checkpoint mechanism inhibits CD8 T cell function in tumor microenvironment [129]. Although immune checkpoint blockade molecules, anti-CTLA-4 antibody and anti-PD-1 antibody, have not been proven currently to have the effectiveness, there is too little information to decide efficacy of ipilimumab and nivolumab in sarcomas. Thus, immune checkpoint blockade medicines should be evaluated in the future. Adoptive cell transfer approaches are also the subject of new sarcoma treatment trials. Overall, these trials and successes suggest that immunotherapy is moving to the forefront of therapy for bone and soft tissue sarcomas.

## Figures and Tables

**Table 1:** Cancer testis antigens in bone and soft tissue sarcomas.

Sarcoma subtypes	Expression of cancer testis antigens						
	NY-ESO	LAGE	MAGE-A3	MAGE-A4	MAGE-A9	PRAME	SSX-2
Bone sarcomas							
Osteosarcoma [14]	+	+	+	+			
Ewing's sarcoma [14]	+	+	+	+			
Chondrosarcoma [14]	+	+	+	+			
Soft tissue sarcomas							
Synovial sarcoma [15]	+	+	+	+	+	+	+
Malignant fibrous histiocytoma, pleomorphic spindle cell sarcoma [15]	+	+	+			+	+
Liposarcoma [15]	+	+	+	+	+		+
Leiomyosarcoma [15]		+	+	+	+	+	

**Table 2:** Clinical trials stimulating innate immunity against bone and soft tissue sarcomas.

Agent	Number of patients	Diagnosis	Treatment	Follow-up	Clinical result
IL-2 [16]	6	Osteosarcoma, Ewing's sarcoma	6–12 × 10^6^ IU/m^2^ for 5 days by every 3 weeks	7–71 months	Complete response (CR): 5 Progressive disease (PD): 5
IFNs [17]	3	Osteosarcoma	2.5–5 × 10^6^ IU/mL twice or thrice weekly	6–8 months	CR: 2 PD: 1
IFN-*α*2 [18]	20	Osteosarcoma, fibrosarcoma, chondrosarcoma, and malignant fibrous histiocytoma	5 × 10^7^ IU/m^2^ thrice weekly	1–3 months	Partial response (PR): 3
IFN-*α* [19]	89	Osteosarcoma	Cohort 1 (70 patients); 3 × 10^6^ IU daily for a month Cohort 2 (19 patients); 3 × 10^6^ IU daily for 3–5 years	10 years	Metastatic free survival: 39% Sarcoma specific survival: 43%
IFN-*β* [20]	158	Osteosarcoma (COSS-80)	1 × 10^5^ IU/kg for 22 weeks	30 months	Disease-free survival +IFN: 77% −IFN: 73% (N.S.)
Pegylated IFN-*α*2b [21]	715	Osteosarcoma (EURAMOS-1)	Methotrexate, adriamycin, and cisplatin (MAP) +/−IFN (0.5–1.0 *μ*g/kg/wk) for 2 years	Median follow-up 3.1 years	Event-free survival +IFN: 77% −IFN: 74% (N.S.)
L-MTP-PE [22]	662	Osteosarcoma (INT 0133)	MAP alone, MAP + L-MTP-PE, MAP + ifosfamide, MAP + ifosfamide + L-MTP-PE	6 years	Overall survival +L-MTP-PE: 78% −L-MTP-PE: 70% Event free survival No significant difference

**Table 3:** Clinical trials stimulating adaptive immunity against bone and soft tissue sarcomas.

Agent	Number of patients	Diagnosis	Treatment	Immune response	Clinical result
Autologous tumor cells [23]	23	Sarcoma	Total 1.0 × 10^7^ cells	Delayed-type hypersensitivity (DTH) positive: 8 patients	Median survival DTH responder: 16.6 months Nonresponder: 8.2 months
Tumor translocation breakpoint specific peptide-pulsed DCs [24]	52	Ewing's sarcoma, rhabdomyosarcoma	Total 4.2–143.0 × 10^6^ cells	39% with immune response to the translocation breakpoint, 25% with response to E7-specific	Overall survival Vaccination: 43% Control: 31%
Tumor-specific synthetic peptides or tumor lysates pulsed DCs [25]	5	Ewing's sarcoma, synovial sarcoma, neuroblastoma	2–15 × 10^6^ pulsed DCs injected 6–8 times	DTH positive: 1 patient	CR: 1 (77 months) PD: 4 (2–27 months)
A 9-mer peptide from SYT-SSX fusion site [26]	21	Synovial sarcoma	0.1 or 1.0 mg peptide +/− adjuvant 6 times at 14-day interval	Tetramer positive CD8: 7 patients	Stable disease (SD): 1/9 peptide alone 6/12 vaccine with adjuvant
Anti-CTLA-4 antibody [27]	6	Synovial sarcoma (expressed NY-ESO-1)	Ipilimumab 3 mg/kg every 3 weeks for 3 cycles	DTH: all patients negative	Time to progression 0.47–2.1 months (median 1.85), overall survival time 0.77–19.7 months (median 8.75)
T cell receptor- (TCR-) transduced T cells (NY-ESO-1 specific) [28]	6	Synovial sarcoma (expressed NY-ESO-1)	TCR-transduced T cells +720,000 IU/kg of IL-2	Tetramer positive CD8: 5 patients	PR: 4 PD: 2
